# Specific cortical and subcortical grey matter regions are associated with insomnia severity

**DOI:** 10.1371/journal.pone.0252076

**Published:** 2021-05-26

**Authors:** Neus Falgàs, Ignacio Illán-Gala, Isabel E. Allen, Paige Mumford, Youssef M. Essanaa, Michael M. Le, Michelle You, Lea T. Grinberg, Howard J. Rosen, Thomas C. Neylan, Joel H. Kramer, Christine M. Walsh

**Affiliations:** 1 Atlantic Fellow for Equity in Brain Health at the University of California San Francisco, Department of Neurology, University of California San Francisco, San Francisco, California, United States of America; 2 Global Brain Health Institute, University of California, San Francisco, California, United States of America; 3 Department of Neurology, Memory & Aging Center, Weill Institute for Neurosciences, University of California, San Francisco, California, United States of America; 4 Alzheimer’s Disease and other Cognitive Disorders Unit, Department of Neurology, Hospital Clínic de Barcelona, Universitat de Barcelona, Barcelona, Catalonia, Spain; 5 Sant Pau Memory Unit, Department of Neurology, Hospital de la Santa Creu i Sant Pau, Universitat Autònoma de Barcelona, Barcelona, Catalonia, Spain; 6 Department of Epidemiology and Biostatistics, University of California San Francisco, San Francisco, California, United States of America; 7 Department of Psychiatry, Weill Institute for Neurosciences, University of California, San Francisco, California, United States of America; 8 Department of Pathology, University of Sao Paulo Medical School, Sao Paulo, Brazil; 9 Department of Pathology, University of California, San Francisco, California, United States of America; 10 Stress & Health Research Program, Department of Mental Health, San Francisco VA Medical Center, San Francisco, California, United States of America; University of Medicine & Dentistry of NJ - New Jersey Medical School, ISRAEL

## Abstract

**Background:**

There is an increasing awareness that sleep disturbances are a risk factor for dementia. Prior case-control studies suggested that brain grey matter (GM) changes involving cortical (i.e, prefrontal areas) and subcortical structures (i.e, putamen, thalamus) could be associated with insomnia status. However, it remains unclear whether there is a gradient association between these regions and the severity of insomnia in older adults who could be at risk for dementia. Since depressive symptoms and sleep apnea can both feature insomnia-related factors, can impact brain health and are frequently present in older populations, it is important to include them when studying insomnia. Therefore, our goal was to investigate GM changes associated with insomnia severity in a cohort of healthy older adults, taking into account the potential effect of depression and sleep apnea as well. We hypothesized that insomnia severity is correlated with 1) cortical regions responsible for regulation of sleep and emotion, such as the orbitofrontal cortex and, 2) subcortical regions, such as the putamen.

**Methods:**

120 healthy subjects (age 74.8±5.7 years old, 55.7% female) were recruited from the Hillblom Healthy Aging Network at the Memory and Aging Center, UCSF. All participants were determined to be cognitively healthy following a neurological evaluation, neuropsychological assessment and informant interview. Participants had a 3T brain MRI and completed the Insomnia Severity Index (ISI), Geriatric Depression Scale (GDS) and Berlin Sleep Questionnaire (BA) to assess sleep apnea. Cortical thickness (CTh) and subcortical volumes were obtained by the CAT12 toolbox within SPM12. We studied the correlation of CTh and subcortical volumes with ISI using multiple regressions adjusted by age, sex, handedness and MRI scan type. Additional models adjusting by GDS and BA were also performed.

**Results:**

ISI and GDS were predominantly mild (4.9±4.2 and 2.5±2.9, respectively) and BA was mostly low risk (80%). Higher ISI correlated with lower CTh of the right orbitofrontal, right superior and caudal middle frontal areas, right temporo-parietal junction and left anterior cingulate cortex (*p*<0.001, uncorrected FWE). When adjusting by GDS, right ventral orbitofrontal and temporo-parietal junction remained significant, and left insula became significant (*p*<0.001, uncorrected FWE). Conversely, BA showed no effect. The results were no longer significant following FWE multiple comparisons. Regarding subcortical areas, higher putamen volumes were associated with higher ISI (*p*<0.01).

**Conclusions:**

Our findings highlight a relationship between insomnia severity and brain health, even with relatively mild insomnia, and independent of depression and likelihood of sleep apnea. The results extend the previous literature showing the association of specific GM areas (i.e, orbitofrontal, insular and temporo-parietal junction) not just with the presence of insomnia, but across the spectrum of severity itself. Moreover, our results suggest subcortical structures (i.e., putamen) are involved as well. Longitudinal studies are needed to clarify how these insomnia-related brain changes in healthy subjects align with an increased risk of dementia.

## Introduction

Insomnia is a frequent sleep disorder defined as recurrent poor sleep quality or quantity due to difficulties in initiating or maintaining sleep (DSM-5). The prevalence of insomnia in older adults varies through epidemiological studies, but it has been estimated to affect up to 20% of the healthy older adult population [1]. In recent years, growing evidence has suggested that poor sleep quality may provide an increased risk for dementia [2,3]. Along this line, insomnia has been associated with the impairment of declarative memory in older adults, also affecting their general functioning [4–6]. Sleep appears to be intertwined with proteinopathies, where poor sleep quality is associated with β-amyloid dysregulation and sleep regulating regions are affected by abnormal protein deposition (i.e, tau, synuclein) early in many neurodegenerative disorders [7–11]. Taken together, this suggests that sleep disorders such as insomnia may be detrimental to brain health, possibly contributing to the development of neurodegenerative diseases [12,13].

Defining structural brain changes associated with insomnia could be helpful to understand the underlying changes conferring a risk for dementia in healthy individuals. This has become a topic of interest in recent years, where studies have investigated differences in grey matter (GM) and subcortical regions between insomniacs and non-insomniac older adults [14–16]. Although there is certain variability on the areas related to the report of insomnia across studies, findings in prefrontal areas, putamen or thalamus are more consistent, suggesting that they might participate in insomnia status [14,15,17,18]. Despite the participation of prefrontal or subcortical regions in insomnia, it remains unclear how these structural brain changes may align with measures of the actual severity of the reported insomnia. For instance, although insomnia is a well-defined sleeping disorder, insomnia symptoms can still happen in non-insomniac individuals. Questionnaires evaluating insomnia severity such as the Insomnia Severity Inventory (ISI) are sensitive to the full range of insomnia symptoms, even if they are below the threshold of insomnia diagnosis [19]. This tool provides the opportunity to correlate the whole severity spectrum of insomnia complaints with their potential underlying brain changes.

Secondary symptoms of insomnia can be caused by sleep apnea and depression, which are common in older adults, and could have potential effects on brain health as well. Therefore, when studying insomnia/brain health correlates in older adults, it is ideal to try to evaluate factors which could themselves alter brain health. Therefore, in the current study we utilize an existing dataset to assess the association of both cortical and subcortical GM regions to perceived insomnia severity in a cohort of healthy older adults, taking into account the potential effect of depression and sleep apnea. We hypothesize that insomnia severity is correlated with 1) cortical regions responsible for regulation of sleep and emotion, such as the orbitofrontal cortex, and 2) subcortical regions, such as the putamen.

## Materials and methods

### Participants

One hundred and twenty healthy subjects over 60 years old were selected from the Hillblom Healthy Aging network at the Memory and Aging Center, UCSF from 2012 to 2020 if they had completed the insomnia severity index questionnaire and had undergone magnetic resonance imaging. The study was approved by the UCSF Institutional Review Board, and all participants gave their written, informed consent. Subjects were determined to be cognitively healthy following a comprehensive neurological evaluation, neuropsychological assessment and informant interview. All subjects scored 0 for Clinical Dementia Rating (CDR).

### Insomnia severity assessment

Subjective insomnia severity was assessed by the Insomnia Severity Inventory (ISI) [19]. The ISI is a self-reported questionnaire evaluating seven insomnia-related component scores: severity of difficulties with sleep onset, sleep maintenance, early morning awakening problems, sleep dissatisfaction, interference of sleep difficulties with daytime functioning, noticeability of sleep problems by others, and distress caused by the sleep difficulties. Following the instructions, participants rated each component from 0 to 5, indicating ‘no problem’ to ‘very severe problem’, respectively. The responses were summed to obtain the total score, which could range from 0 to 28.

### Berlin Sleep Questionnaire for sleep apnea

The presence of obstructive sleep apnea was evaluated by the Berlin Questionnaire for sleep apnea (BA) [20]. The questionnaire consists of three categories related to the risk of having sleep apnea, including snoring and breathing, sleepiness, blood pressure and body mass index. The final score classifies individuals into High Risk or Low Risk for sleep apnea.

### Geriatric Depression Scale

The presence of depressive symptoms was assessed by the Geriatric Depression Scale (GDS) [21]. The GDS is a 30-item questionnaire where participants respond to yes/no questions how they felt over the past week. Scores of 0–4 are considered normal, depending on age, education, and complaints; 5–8 indicate mild depression; 9–11 indicate moderate depression; and 12–15 indicate severe depression.

### Brain MRI imaging

#### Acquisition

MRI scans were examined at the UCSF Neuroscience Imaging Center on a Siemens Trio 3.0 T scanner (n = 86) or 3.0 T Prisma scanner (n = 34). T1-weighted magnetization-prepared rapid gradient-echo (MP-RAGE) structural scans were acquired with sagittal orientation, slice thickness = 1.0 mm; slices per slab = 160; plane resolution = 1.0x1.0 mm; matrix = 240X256, repetition time = 2300 ms, echo time = 2.98 ms, inversion time = 900 ms, flip angle = 9°.

#### Neuroimage processing

MRIs were processed with the CAT12 toolbox (http://www.neuro.unijena.de/cat/, version 1450) within SPM12 (http://www.fil.ion.ucl.ac.uk/spm/software/spm12/, version 7487, running in MATLAB r2019b) and we recorded the IQR score during standard preprocessing as a measure of overall quality of images [22]. CTh estimates obtained with CAT12 are accurate and robust and can be considered a fast and reliable alternative to other approaches for the analysis of cortical thickness [23]. The CAT12 toolbox uses tissue segmentation to estimate the white matter distance, and it then projects the local maxima (which is equal to CTh) to other GM voxels by using a neighbor relationship described by the white matter distance. Topological correction, spherical mapping, and spherical registration were performed to obtain vertex wise CTh. We then calculated the mean CTh at each region in the Desikan atlas and the volumes of subcortical GM structures in the neuromorphometrics atlas, as implemented in CAT12.

#### Statistical analyses of clinical outcomes

To analyze the association of insomnia symptoms with depressive symptoms and sleep apnea, we performed pairwise correlations on ISI scores with GDS and BA scores.

#### Statistical analyses of ISI and cortical thickness

To study the correlations between CTh and ISI we performed linear modeling of the CTh maps as implemented in CAT12. We included age, sex, handedness and scan type as covariates. We applied the correction for multiple comparisons using Family-wise error (FWE) with a threshold of *p*<0.05 for cluster significance. This model was repeated to include BA as a covariate, and again to include GDS as a covariate. Additionally, we further performed CTh *t*-test sub-analyses to identify CTh differences between the insomnia (ISI≥8) and non-insomnia groups (ISI ≤7), adjusting by age, sex, handedness, scan type and GDS.

#### Statistical analyses of ISI and subcortical volumes

Because subcortical gray matter volumes (but not the CTh) depend on total intracranial volume (TIV), we divided subcortical gray matter volumes by TIV of each participant to obtain normalized subcortical volumes. Using Stata 16.1 (College Station, Texas), we performed a multivariable linear regression for each subcortical volume to identify significant predictors of ISI while adjusting for age, sex, handedness and scan type. This model was repeated to include BA as a covariate, and again to include GDS as a covariate. We applied the correction for multiple comparisons using False Discovery Rate (FDR). In addition, we performed *t*-test sub-analyses between insomnia (ISI≥8) and non-insomniaa (ISI ≤7) groups.

## Results

### Demographics

Detailed demographics data are shown in Table 1. Participants ranged in age from 62 to 88 years old. 55.7% of the sample were female and all participants had at least 12 years of education. The ISI total scores ranged from 0 to 16 corresponding to ‘non-insomnia’, ‘sub-threshold insomnia’ and ‘moderate insomnia’ categories.

**Table 1:** Cohort descriptors.

	Healthy subjects (n = 120)
**Age**	74.8±5.7 (62–88)
**Gender (% women)**	55.7%
**Years of education**	17.4±2.1 (12–20)
**MMSE**	29±1.1 (26–30)
**ISI total score**	4.9±4.2 (0–16)
**ISI score categories (%)**	
No clinically significant insomnia (0–7)	71
Subthreshold insomnia (8–14)	26
Clinical insomnia (moderate severity) (15–21)	3
Clinical insomnia (severe) (22–28)	0
**GDS score**	2.5±2.9 (0–15)
**BA score (% low risk)**	80.2

Data are presented as means ± standard deviation (range). MMSE, Mini Mental State Examination; ISI, Insomnia Severity Index; GDS, Geriatric Depression Scale; BA, Berlin Apnea index.

### Association between ISI scores and other clinical outcomes

**Fig 1:**
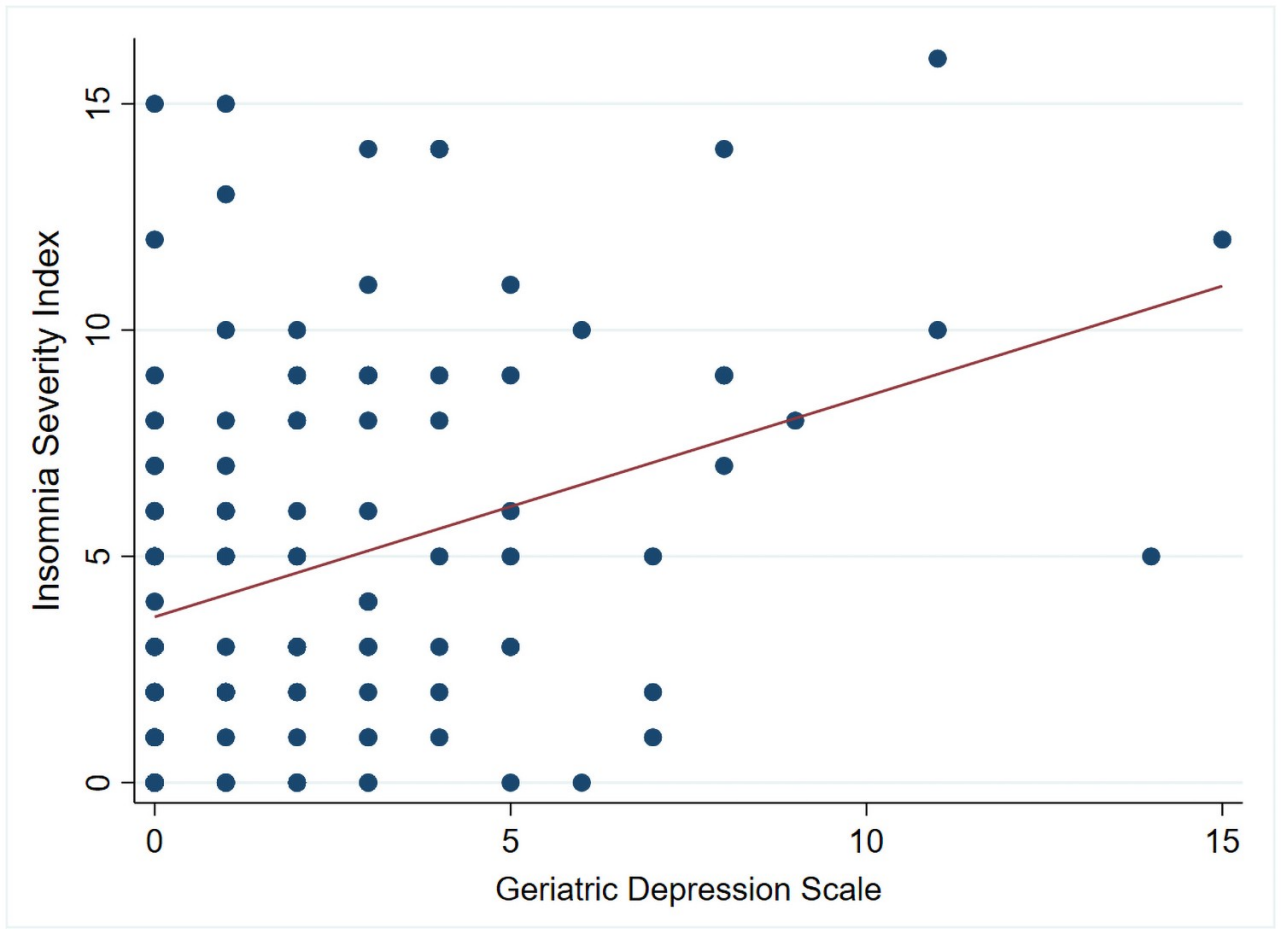
Correlation between Insomnia Severity Index and Geriatric Depression Scale. The figure shows a moderate positive correlation between ISI scores and Geriatric Depression Scale (r = 0.34, *P*-value < .001) in all participants (n = 119).

ISI and GDS scores showed a significant positive correlation (r = 0.34, *p*<0.01) (Fig 1) while BA did not.

### Associations between ISI scores and GM regions

#### Cortical regions

**Fig 2:**
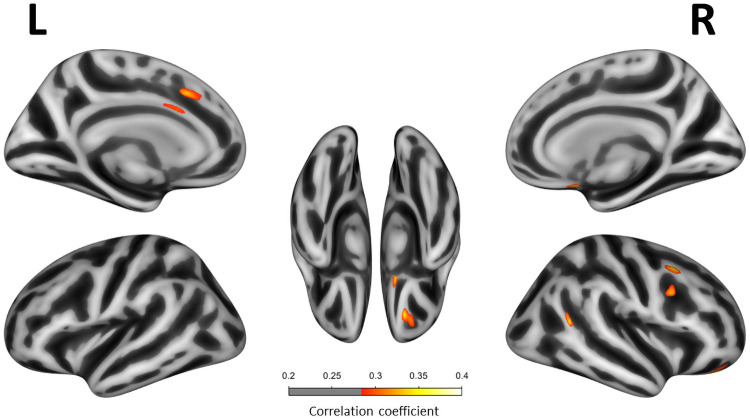
Correlation between Insomnia Severity Index scores and cortical thickness. Correlation between ISI scores and cortical thickness in all participants (n = 120). Only regions with *P*-value < .001 (uncorrected FWE) are shown. All multivariate linear regression models were adjusted for age, sex, handiness and scan type. Correlation coefficients are expressed as a color scale, indicating an increasing strength of the correlation from red to yellow.

**Fig 3:**
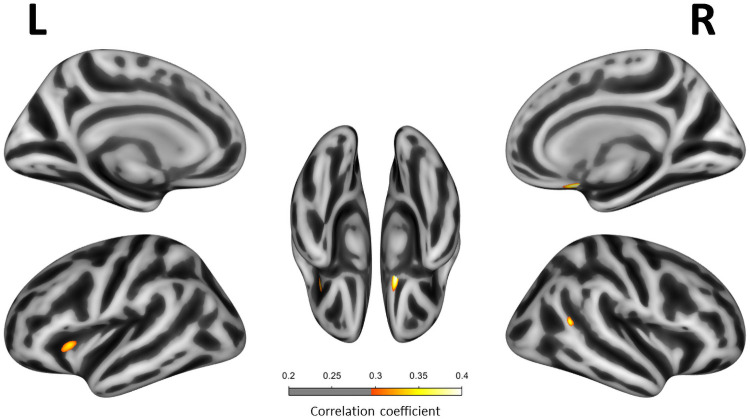
Correlation between Insomnia Severity Index scores and cortical thickness adjusting by Geriatric Depression Scale. Correlation between ISI scores and cortical thickness in all participants (n = 119). Only regions with *P*-value < .001 (uncorrected FWE) are shown. All multivariate linear regression models were adjusted for age, sex, handiness and scan type and Geriatric Depression Scale. Correlation coefficients are expressed as a color scale, indicating an increasing strength of the correlation from red to yellow.

Multivariate analyses adjusting for age, sex, handedness and scan type showed that higher ISI scores correlated with lower CTh in right orbitofrontal, right superior and caudal middle frontal areas, right temporoparietal junction and left anterior cingulate regions (*p*<0.001, uncorrected FWE) (Fig 2). Covarying for BA did not alter the findings. When covarying for GDS, the negative correlations in the right orbitofrontal and temporo-parietal junction remained unchanged. The correlations in the right superior and caudal middle frontal and left anterior cingulate regions, however, were lost. Further, we found that, when covarying for GDS, ISI was negatively correlated with CTh in the left insula (*p*<0.001, uncorrected FWE) (Fig 3). None of these clusters remained significant after the FWE multiple comparison correction. The CTh *t*-test comparisons between insomnia and non-insomnia groups showed lower CTh in certain orbitofrontal, prefrontal and temporo-parietal regions (p<0.001, uncorrected). However, differences in the insula were not replicated. Detailed results of the *t*-test sub-analyses are shown on S1 Fig.

#### Subcortical regions

**Fig 4:**
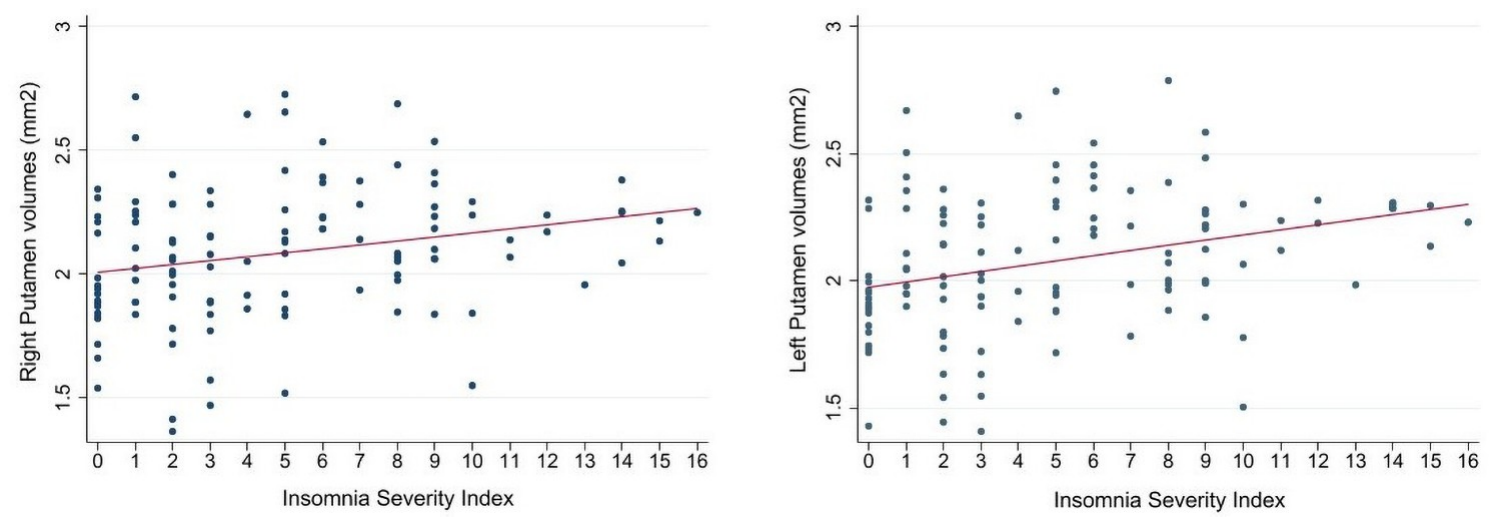
Correlation between Putamen volumes and Insomnia Severity Index. Scatter plot showing the correlation of the normalized volumes of right and left putamen with ISI scores (n = 120).

The detailed results from the linear regressions assessing subcortical volumes and ISI scores adjusted by age, sex, handedness and scan type are shown in Table 2. Right and left putamen showed a significant correlation with ISI, where greater putamen volumes were associated with higher ISI scores (*p*<0.01 uncorrected, Fig 4). Only the correlation with left putamen volumes survived FDR multiple comparisons (*p*<0.05). Covarying for GDS and BA did not alter the findings. Additionally, box plot graphs showing the mean normalized volumes of the right and left putamen in insomnia (ISI≥8) and non-insomnia (ISI ≤7) subjects are shown in S2 Fig.

**Table 2:** Effect of subcortical regional volumes to ISI scores.

Region of interest	β	*p*
**Right Caudate**	0.08	.405
**Right Putamen**	0.22	.016^a^
**Right Thalamus**	0.07	.525
**Right Pallidum**	0.48	.608
**Left Caudate**	0.10	.303
**Left Putamen**	0.26	.005^a^
**Left Thalamus**	0.07	.531
**Left Pallidum**	0.09	.328

Data are presented standardized beta (β). This table shows the effect of subcortical gray matter brain regions to ISI scores using linear model effects adjusting by age, sex, handiness, MRI scanner and total intracranial volume (FDR uncorrected).

^a^*p* significant values (*p*<0.05).

## Discussion

We performed a cross-sectional study analyzing the relationship between cortical and subcortical grey matter regions and the degree of perceived insomnia severity in a cohort of healthy older adults. We found that lower volumes in certain cortical areas (i.e., right ventral orbitofrontal, right temporo-parietal junction and left insula) and greater volumes in subcortical structures (i.e., putamen) were related to higher perceived insomnia severity independent of the presence of depression or likelihood of sleep apnea. Hence, identifying a detrimental relationship between a insomnia symptoms and CTh in otherwise healthy aging populations.

Compared to prior studies reporting insomnia prevalence around 30–35% in healthy aging populations, our cohort presents relatively low ISI scores that fall into the “no clinically significant insomnia” to “subthreshold insomnia” categories [1,14]. These differences might be related to methodological approaches and sample characteristics. Methodological differences involving tools or diagnostic criteria used to identify insomniacs can modify prevalence estimates, through differences in sensitivity and specificity [14,19]. Most of the studies assessing sleep quality in healthy aging cohorts are focused on younger populations over 50 years old, while our cohort is over 62 years old and could account for some of the difference. It is also possible that socio-economic statuses or prior education could influence the incidence of reporting insomnia, e.g. our cohort has a relatively high mean education of 17.4 years, with many participants having masters or doctorate degrees. Education, a proxy for cognitive reserve, might potentially shape the odds for insomnia development later in life as well.

Nevertheless, despite the low insomnia severity found in our cohort, we still found an association between ISI and cortical thickness. This fact is particularly remarkable because it reinforces the observation that mild-moderate insomnia symptoms could have effects on brain structure in healthy aging populations, even with individuals below the threshold of insomnia diagnosis. Overall, this emphasizes the importance of the whole spectrum of insomnia severity with brain health in aging. Our findings highlighted the orbitofrontal cortex as a main cortical region associated with the severity of insomnia even when adjusting by depressive symptoms and sleep apnea. Specifically, we found smaller cortical volumes in the ventral orbitofrontal were associated with worse levels of insomnia. This is congruent with prior publications that have detected lower volumes of orbitofrontal cortex in insomnia subjects, as well as in healthy individuals with sleep fragmentation and early-morning awakenings [14,24–27]. Similarly, a report on a small sample of older adults (mean age 60 ± 6, age range 52–74) found that smaller orbitofrontal volumes were associated with higher scores in the insomnia subscale of the Sleep Disorders Questionnaire [15]. Our findings support the role of orbitofrontal regions in insomnia but furthermore, extend this, confirming that the gradient of insomnia severity is associated with volumetric values in these areas. As part of the highly interconnected limbic system, the orbitofrontal cortex participates in decision-making processes and emotion signaling, suggesting its dysfunction could lead to increased insomnia mediated by mood alterations. However, the orbitofrontal cortex also has a role in evaluating thermal comfort [26]. Insomnia patients have difficulties judging thermal comfort, and interestingly, small changes from comfortable temperatures have a detrimental effect on sleep quality [28]. Therefore, insomnia could also result from orbitofrontal dysfunction leading to the inability to recognize the optimal temperature for sleep [26,28].

In addition, we also found that smaller areas of the dorsolateral prefrontal cortex were associated with worse levels of insomnia. The dorsolateral prefrontal cortex has long been implicated in high level cognitive functions such as attention, working memory, decision-making and reasoning. Impaired prefrontal functioning such as problem-solving abilities and emotional processing are associated with mood disorders (e.g. depression) that frequently are related or can lead to insufficient sleep [29]. Our findings support the role of the dorsolateral prefrontal cortex on insomnia symptoms especially when depressive symptoms may be contributing. In alignment with the hypothesis of emotional processing as a key character for insomnia development, a recent report highlighted right prefrontal areas (i.e, orbitofrontal) as a neuropathological core mechanism for the intersection of insomnia with mood symptoms (i.e, depression) [30]. Interestingly, our findings show this right-lateralized association between perception of insomnia severity not only in orbitofrontal but also in dorsolateral areas, reinforcing the influence of emotional processing and mood to the perception of mild insomnia symptoms.

However, the relationship between insomnia and brain atrophy might work in the opposite direction, meaning not only that grey matter volume may contribute to insomnia, but also insomnia could have an effect on grey matter structure. In this line, prior studies have reported morphological brain changes after sleep deprivation [31,32]. This suggests that insufficient sleep could modulate certain neurobiological processes that could potentially affect cortical gray matter structure (e.g. metabolite clearance, synaptic homeostasis, gene expression, macromolecule biosynthesis, neuroinflammation, oxidative stress) [33–35]. Interestingly, the prefrontal cortex is especially susceptible to oxidative stress and furthermore, sleep deprivation especially affects neuropsychological performance on tasks related to the prefrontal cortex (executive domains) [36]. Thus, it is possible that grey matter health in dorsal-orbitofrontal areas is vulnerable to insufficient sleep [37,38].

In addition, other cortical areas such as anterior cingulate have been associated with insomnia severity in the present study although its effect disappeared when controlling for depression. The cingulate cortex is considered part of the limbic system and participates in many high functions such as learning, memory processing, and emotion. It is plausible that the cingulate cortex/insomnia relationship is driven by the effect of mood disorders (i.e; depression). Although its role in sleep regulation is still poorly defined, it has been suggested to also participate in specific processes such as the mediation of slow-waves during slow-wave sleep [39]. Most of the prior studies described lower volumes of the anterior or posterior cingulate in insomniacs [14,40,41]. Our findings are congruent with these prior reports, extending the literature to show that these cingulate areas could be associated with insomnia severity. However, one previous study did report that larger rostral anterior cingulate volumes were associated with worse sleep in chronic insomnia patients [42]. Since chronic insomnia increases the risk for depression, these findings were interpreted as a compensatory response to repetitive sleep disturbance and a possible marker of resilience to developing mood disorders. The Winkelmann study did not study older adults, but was restricted to young-middle-aged patients (mean age 39.3 ±8.7), which accounts for the conflicting results, and could suggest that there is an effect of aging on diminished resilience. Alternatively, the Winkelmann compensatory finding could be restricted to those with chronic insomnia, as the insomnia severity in our population was mild-moderate.

On the other hand, the inferior parietal lobe together with the overlapping temporoparietal junction participate in a broad range of behaviors and functions such as attention, language processing, social cognition and self-awareness. As these are areas highly connected to diverse functional networks, they are thought to act more as a hub for multimodal integration, with probable participation in many cognitive processes [43]. Although the direct participation of this area in sleep might be controversial, we believe that its involvement in insomnia is conceivable, specifically in the context of self-perception of sleep disturbance. In line with this hypothesis, a longitudinal study demonstrated that older adults with poor sleep quality developed a widespread pattern of atrophy including these temporal-parietal areas as well [44].

Lastly, our cortical findings showing lower insular volumes are associated with insomnia severity, but only when withdrawing the effect of the depressive symptoms, is surprising. The insula is highly related to emotional processing and mood disorders, so we would expect it to be more related to the depressive symptoms than directly to insomnia. Indeed, the supplementary analyses comparing insomnia vs non-insomnia groups within our cohort did not replicate this difference in the insula, supporting this hypothesis. However, prior literature has demonstrated changes in functional connectivity in the anterior insula after sleep deprivation [45,46]. Moreover, lesions on the anterior insula in rats elicits decreased wakefulness and increased rapid eye movement (REM) sleep and non-REM (NREM) sleep, suggesting that the insula could participate more than we thought in sleep-wake regulation processes [47].

The cortico-striato-thalamo-cortical loop has multiple neurocognitive functions including regulating arousals along with cognitive and affective functions. The striatum, formed by putamen and caudate, is specially involved in sleep regulation [17]. In physiological terms, putamen regulates arousal by inhibitory GABAergic projections to pallidum and thalamus, promoting cortical activity and wakefulness [48]. In the same line, bilateral lesions of the putamen have demonstrated to reduce the time spent in wakefulness [49]. Furthermore, putamen is involved in motor regulation via connections with the primary motor cortex and premotor areas regulating restlessness, a manifestation of physiological arousal [50]. Morphometric studies that evaluated subcortical structures in insomnia patients, suggested altered volumes of putamen or thalamus in this population [14,18,51]. However, results are not consistent between studies showing either positive or negative correlations with sleep parameters. Our results support the effect of greater putamen volumes on insomnia severity, in line with its suggested wake-promoting role. The putamen, as a main component of the striatum, is highly interconnected with frontal cortical areas such as the orbitofrontal and prefrontal regions. The connectivity between these cortico-subcortical regions shapes the frontostriatal circuit, which has been related to sleep-wake regulation. Although the mechanism by which subcortical areas, such as the putamen, regulate arousal is still poorly understood, the dysfunction of the frontostriatal circuit seems to have a role in insomnia. In this line, prior functional MRI studies have reported altered patterns of connectivity between subcortical (putamen) and cortical (frontal) regions in insomnia patients [52–54]. We found no relationship, however, with thalamic volumes. Although it is known that subcortical structures participate in sleep regulation, further studies specifically evaluating which subcortical changes directly relate to insomnia are needed [17].

Aging is associated with neuronal dysfunction in terms of metabolic, proteostasis impairment and oxidative stress that can trigger amyloid-β, tau, and α -synuclein accumulation. The poor sleep quality and sleep deprivation associated with insomnia complaints, could accelerate these age-related changes potentiating disease-specific neuronal vulnerabilities causing neurodegenerative disorders [55]. For instance, results from a recent study suggest that that sleep disturbances could predict accumulation of beta-amyloid across subsequent years [11]. Furthermore, relevant changes within frontal regions, from cortical atrophy to protein accumulation, have been identified in many early neurodegenerative disorders [27,56,57]. This evidence aligns with our findings reinforcing the idea of these areas related to insomnia severity, as especially vulnerable to neurodegenerative disease processes. Other sleep disorders have also been associated with subsequent onset of cognitive or neurodegenerative disorders. For example, periodic limb movements (PLMS), which precede dysexecutive impairment and REM sleep behavior disorder (RBD), has been shown to predict the development of Parkinson’s Disease [58,59]. Further investigations are required to understand the relationship between neurodegenerative processes and various sleep disorders, including PLMS, RBD, and now insomnia.

Even though our results show an existing correlation between insomnia severity and certain gray matter changes, they do not confirm its causality. The relationship observed between both parameters could indicate that these specific gray matter changes are driving the severity of insomnia or, that insomnia severity has a detrimental effect on these areas. Ultimately, these brain changes could be happening in parallel with insomnia severity without being directly caused by it.

The main strengths of this study are the well-characterized participants as cognitively healthy, older individuals. It is possible that cognitively healthy older adults with insomnia could be at greater risk for developing a neurodegenerative disease. However, we did not limit our cohort to those with high levels of insomnia. Evaluating not only individuals meeting criteria for insomnia disorder but also patients with slight insomnia symptoms, provides awareness of the effect the entire clinical spectrum of insomnia has on brain health. An important limitation of the present study is that the findings are restricted to subjective measures of insomnia as opposed to objective measures. Since BA identifies individuals at high risk for sleep apnea but does not confirm its actual presence, the contribution of sleep apnea to brain changes might be underestimated. Therefore, further studies assessing the presence of sleep apnea with objective (at-home or in-lab) diagnostic methods are needed. Additionally, individuals in our sample are cognitively intact but we do not have measures of neuropathological burden to detect silent underlying neurodegenerative changes. In this line, age-related changes such as vascular damage or the dysfunction of other subcortical nuclei within the brainstem, not evaluated in our study, could potentially modulate the observed relationship between cortical thickness and insomnia. A further limitation of the study is that the CTh correlations did not survive the correction for multiple comparisons. Although that was expected due to the small effect size of insomnia severity on CTh in an otherwise healthy population it could hamper the interpretation of the data. Further studies evaluating the relationship between insomnia and structural brain changes are warranted.

## Conclusions

In conclusion, certain cortical areas (e.g, orbitofrontal, insula, temporo-parietal junction) and subcortical areas (i.e, putamen) are associated to the perception of higher insomnia severity, even in those individuals with mild insomnia symptoms and considering the added effect of insomnia-related comorbidities as depressive symptoms and sleep apnea.

Further longitudinal studies are needed to clarify how these insomnia-related brain changes in healthy subjects align with an increased risk of dementia.

## Supporting information

S1 Fig Cortical thickness differences between insomnia and non-insomnia groups, adjusted by Geriatric Depression Scale. Cortical thickness t-test comparison between insomnia *(ISI≥8)* and non-insomnia *(ISI ≤7)* groups (n = 119) adjusting by age, sex, handedness, scan type and Geriatric Depression Scale. Only regions with *P*-value < .001 (uncorrected FWE) are shown. T-values are expressed as a color scale.
(TIF)

S2 Fig Putamen volumes between insomnia and non-insomnia groups. Box plot showing the mean of the normalized volumes of right and left putamen in insomnia *(ISI≥8)* and non-insomnia *(ISI ≤7)* subjects (n = 120). *Significance p<0.05.
(TIF)
