# Supplementary figures and images for: Specific cortical and subcortical grey matter regions are associated with insomnia severity
Source: PLoS One. 2021 May 26;16(5):e0252076. doi: 10.1371/journal.pone.0252076 (PMC8153469; doi:10.1371/journal.pone.0252076)

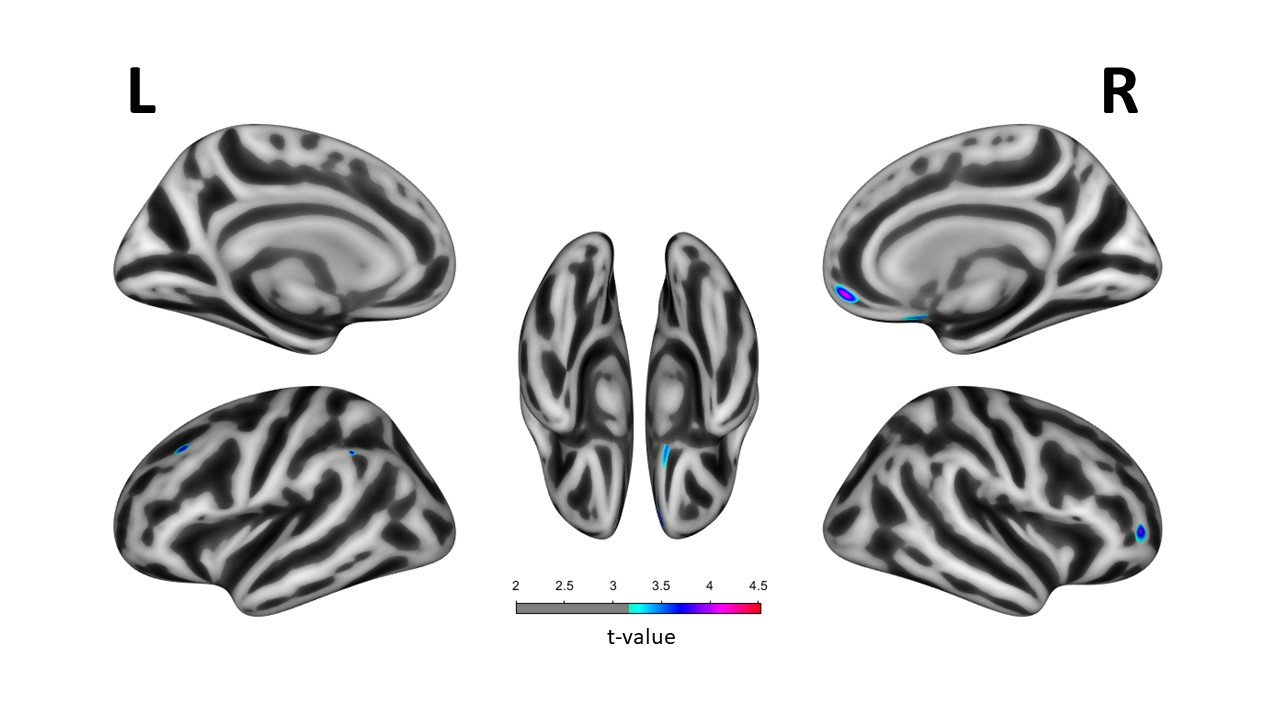

Supplement: S1 Fig — Cortical thickness t-test comparison between insomnia (ISI≥8) and non-insomnia (ISI ≤7) groups (n = 119) adjusting by age, sex, handedness, scan type and Geriatric Depression Scale. Only regions with P-value < .001 (uncorrected FWE) are shown. T-values are expressed as a color scale. (TIF) [file pone.0252076.s001.tif]

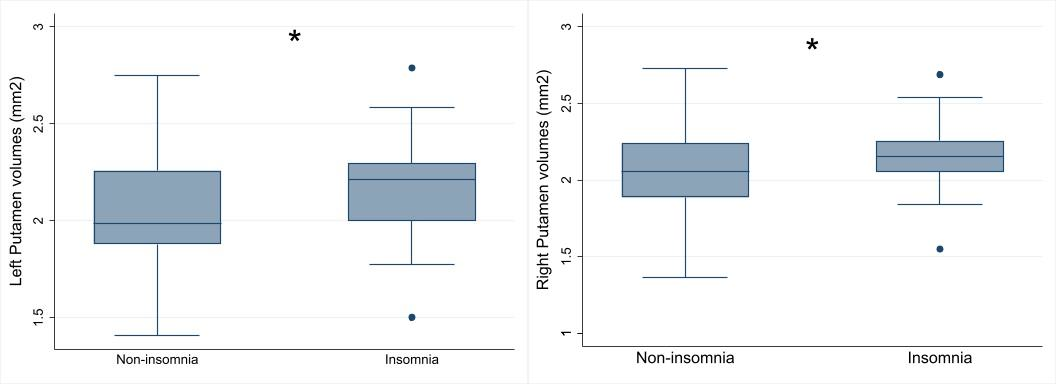

Supplement: S2 Fig — Box plot showing the mean of the normalized volumes of right and left putamen in insomnia (ISI≥8) and non-insomnia (ISI ≤7) subjects (n = 120). *Significance p<0.05. (TIF) [file pone.0252076.s002.tif]
