# Supplementary material for: Specific cortical and subcortical grey matter regions are associated with insomnia severity
Source: PLoS One. 2021 May 26;16(5):e0252076. doi: 10.1371/journal.pone.0252076 (PMC8153469; doi:10.1371/journal.pone.0252076)
Supplement: Attachment — Submitted filename: Response to reviewers .docx [file pone.0252076.s003.docx]

We thank the reviewers for their time and insightful comments for our manuscript: “Specific cortical and subcortical grey matter regions are associated with insomnia severity” (PONE-D-20-36484). We appreciate Reviewer 1 highlighting the importance of the study and the resulting manuscript. We have addressed both the Reviewer’s and Editor’s comments below and in the manuscript. We hope they alleviate any of your prior concerns. Your comments have improved the clarity of our manuscript.

**Response to reviewers:**

**Reviwer #1:**

1. Of all self-report measures, the likelihood of sleep apnea presents the greatest concern as both insomnia severity and depressive symptoms are diagnosed and treated based on self-report. It could be helpful to better articulate this limitation in the discussion section, and the need for future studies to assess actual presence of apnea via in-lab or at-home overnight testing.

*Thank you for pointing this out. We agree with the reviewer that determining the presence of sleep apnea with objective measures would increase the accuracy of this measurement. We have stressed this limitation in the discussion section and highlighted the need for further studies using objective tools for apnea testing.*

*Line 463-464: Therefore, further studies assessing the presence of sleep apnea with objective (at-home or in-lab) diagnostic methods are needed.*

2. The percentage of individuals with likelihood of apnea seems to be fairly similar to other published healthy older adult samples. However, the insomnia severity and potentially the depressive symptom averages appear lower than usual. It could be helpful to not only provide the average for these measures, but percentage of individuals within each cut-off range. For example, it could be helpful to provide the number of individuals in the “non-insomnia,” “sub-threshold insomnia,” and “moderate insomnia” categories as well as more fully acknowledge the potential differences in lower rates of symptoms in the current sample in the discussion section more fully.

*We agree that providing the categorical distribution of the ISI could be helpful for the reader to better understand the sample characteristics. We have now included the percentage of subjects in each of the categories in Table 1. Furthermore, we have stressed the fact that the subjects included scored mostly in the “no clinically significant insomnia” or “subthreshold insomnia” categories in the discussion section. Moreover, following the reviewer suggestion, we have included the discussion of potential differences in insomnia prevalence between other studies and ours (lines 308-319).*

3. Many of the results do not survive corrections for multiple comparisons. It could be helpful to discuss this more fully in the discussion section and insure it is represented in the study abstract.

*Although it is common in studies of healthy aging utilizing MRI-imaging to observe findings not surviving multiple comparisons due to the small effect size, we fully agree with the reviewer that it is important to highlight this because of its potential implications. We have now addressed this point by extending the statement in the discussion (lines 468-471). Regarding the abstract, we apologize if that was not clear enough by reporting the results as p<0.001, uncorrected FWE. We have now included the statement ‘**The results were no longer significant following FWE multiple comparisons’ (Lines 51-52).*

4. There is possibly a typo in the results sections under the title, “Associations between ISI scores and GM regions”. There is mention of right medial orbitofrontal regions of significance, whereas, otherwise in the paper there is mention of right ventral orbitofrontal regions of significance.

*Thank you for catching this. It was a typo which we have now corrected.*

5. Was this a planned analysis prior to data collection or an exploratory analysis with existing data? It could be helpful to the reader to understand the degree of exploratory approach for the current paper.

*This was a hypothesis driven analysis of existing data. Dr. Walsh was interested in better understanding the association between disrupted sleep and brain health in older adults. Dr. Falgàs with her interests in brain imaging and sleep disruption raised came to Dr. Walsh with her hypotheses in this area and assessed the existing dataset.*

*Line 100-101: Therefore, in the current study we utilize an existing dataset to assess the association of both cortical and subcortical GM regions to perceived insomnia severity in a cohort of healthy older adults, taking into account the potential effect of depression and sleep apnea.*

**Editor comments:**

1. The authors make an important observation relating cortical thickness to a sleep disorder and this point should be further underscored in the presentation of the revised manuscript.

*Thank you for raising this point; we have now addressed the manuscript to highlight this relationship in the manuscript further (305-306, 441-446).*

2. The possible significance of brain cell alterations in the putamen are not sufficiently (if at all) described and explained. For example, what cortical inputs to the putamen could account for such changes? Moreover, in view of its potential importance, it would be helpful to show an MRI indicating how the putamen in question compares to a normal (control) individual. In addition, the authors should make an effort to further explain why or how sleep loss would affect specific regions of cortex and not others.

*We have now extended the explanation on the putamen’s relevance and cited the corresponding literature.*

*Lines 420-427:* The putamen, as a main component of the striatum, is highly interconnected with frontal cortical areas such as the orbitofrontal and prefrontal regions. The connectivity between these cortico-subcortical regions shapes the frontostriatal circuit, which has been related to sleep-wake regulation. Although the mechanism by which subcortical areas, such as the putamen, regulate arousal is still poorly understood, the dysfunction of the frontostriatal circuit seems to have a role in insomnia. In this line, prior functional MRI studies have reported altered patterns of connectivity between subcortical (putamen) and cortical (frontal) regions in insomnia patients [52-54].

*Lu FM et al., Disrupted Topology of Frontostriatal Circuits Is Linked to the Severity of Insomnia. Front Neurosci. 2017 Apr 19;11:214. doi: 10.3389/fnins.2017.00214.*

*Zhou F et al. Altered long- and short-range functional connectivity density associated with poor sleep quality in patients with chronic insomnia disorder: A resting-state fMRI study. Brain Behav. 2020 Nov;10(11):e01844. doi: 10.1002/brb3.1844.*

*Zou G, et al. Altered thalamic connectivity in insomnia disorder during wakefulness and sleep. Hum Brain Mapp. 2021 Jan;42(1):259-270. doi: 10.1002/hbm.25221.*

*When performing MRI-analyses, different factors can contribute to the final measure of the putamen volumes. Because of that, when evaluating the differences in a correlation model or t-test, we control for factors such as Total Intracranial Volume, gender, age, handedness, etc. Although we could provide the image of an MRI-scan of a random patient and control (see below), we consider this comparison not accurate enough to be included in the manuscript. We believe the visual inspection of the putamen on an MRI could be misleading since the direct comparison of the MRI-scan would not be reflecting the complexity of the whole analyses, and it represents a non-adjusted output. However, to provide further information regarding the putamen volumes in insomnia at the group level, we have included Supporting Information Figure 2 (S2 Fig), the boxplots comparing the normalized putamen volumes in insomnia and non-insomnia subjects.*

Control

Insomnia

**S2 Fig. Putamen volumes between insomniacs and non-insomniacs.** Box plot showing the mean of the normalized volumes of right and left putamen in insomnia *(ISI<7)* and non-insomnia *(ISI≤8)* subjects (n=120). *Significance p<0.05

3. It would be helpful if the authors included a control group of subjects with their MRI's (i.e., subjects without sleep disorders but of the same age, medical treatment history, etc.). Otherwise, one may have no idea whether variations in the appearance of the MRI's might not represent normal variations among individuals, especially of middle age and beyond. Such comparisons would strengthen the reliability of the observations.

*Thank you for bringing this up. The present study aims to evaluate the correlation of the spectrum of insomnia severity (using ISI, which scores from 0 to 28) and Cortical Thickness in a cohort of healthy older adults. The reason for this approach is that prior MRI studies have already used a case-control study design (insomniacs vs. controls) (Grau-Rivera et al., 2020; Altena et al., 2010; Sexton et al., 2014). Although these studies have varying results, overall, they highlight a detrimental effect of insomnia or low sleep quality on the orbitofrontal and prefrontal areas. However, insomnia symptoms occur on a spectrum, and focusing on cut-offs or thresholds of insomnia being present vs not present miss out on the importance of addressing even low-level insomnia, which appear to relate to brain changes as well. Therefore, we believe there is an unmet gap in evaluating how the spectrum of insomnia (including symptoms below the insomnia category or mild insomnia symptoms) affects brain changes in healthy aging which we are addressing in this manuscript.*

*The CTh-neuroimaging analyses performed in the present study include the standard normalization step of the MRI-scans to a template during the pre-processing of the images that adjust and, therefore, limit the potential contribution of interindividual variations. Additionally, the regression model adjusted the data by other factors that could potentially influence the results: age, gender, scan type, and handedness. Furthermore, we included GDS (depression scale) and BA (apnea likelihood) because they are frequently associated with insomnia.*

*In order to address your concerns, we have included a comparison of insomniacs (ISI<7) vs non-insomniacs (ISI≤8) in the Supporting Information (S1 Fig).*

**S2 Fig. Cortical Thickness differences between insomniacs and non-insomniacs, adjusted by Geriatric Depression Scale.** Cortical thickness *t*-test comparison between insomnia *(ISI<7)* and non-insomnia *(ISI≤8)* groups (n=119) adjusting by age, sex, handedness, scan type and Geriatric Depression Scale. Only regions with *P*-value <.001 (uncorrected FWE) are shown. T-values are expressed as a color scale.

*Grau-Rivera O, et al. Association between insomnia and cognitive performance, gray matter volume, and white matter microstructure in cognitively unimpaired adults. Alzheimers Res Ther. 2020;12(1):4. Published 2020 Jan 7. doi:10.1186/s13195-019-0547-3.*

*Altena E, et al. Reduced orbitofrontal and parietal gray matter in chronic insomnia: a voxel-based morphometric study. Biol Psychiatry. 2010 Jan 15;67(2):182-5. doi: 10.1016/j.biopsych.2009.08.003.*

*Sexton CE, et al. Poor sleep quality is associated with increased cortical atrophy in community-dwelling adults. Neurology. 2014;83(11):967–973. doi:10.1212/WNL.0000000000000774.*

4. In addition to the correlational analyses, I am curious why the study was not designed to utilize t-tests or ANOVA's to compare the groups in question with appropriate control groups. I believe that such analyses would strengthen the manuscript.

*The study's design was based on the objective of evaluating the correlation of the spectrum of insomnia severity and Cortical Thickness in a cohort of healthy older adults. We used a cohort of older adults enriched by individuals not reaching the insomnia diagnosis level or having mild insomnia to test its correlation with brain Cortical Thickness. We apologize if the correlation results are not clear enough. In order to provide more clarity and to address your concerns, we have performed additional analyses dividing the cohort into two groups: insomnia and controls (non-insomnia). The results of the additional analyses, shown in the S1 Fig, indicate the t-value on the regions showing a statistical difference in Cortical Thickness between insomnia and non-insomnia groups*. *Similar to the correlation analyses, the CTh t-test comparisons between insomnia and non-insomnia groups showed lower CTh in certain orbitofrontal, prefrontal and temporo-parietal areas (p<0.001, uncorrected). However, differences in the insula were not replicated (lines 239-242, 401-403).*

5. It is very difficult to discern from the MRI's shown in the manuscript how one group differs from another. That is why specific comparisons between groups would be helpful

*The brain surface maps shown in figures 2, 3 and Supporting Information Fig 1, are the standard output obtained by the MRI-Cortical Thickness analysis. In Figure 2 and 3, brain maps show the areas on which the correlation between ISI and CTh is statistically significant. In Supplemental Fig 1, brain maps show the areas on which the t-test analyses comparing CTh measures between case (insomnia) and controls (non-insomniacs) are statistically significant. The color scale indicates the strength of the effect, indicating the correlation coefficient for the correlation analyses and the t-value for the t-test. Thank you for bringing this up so we had the opportunity to more fully explain how to interpret the figures in the figure legends; we hope the analyses output are now clearer for the reader (lines 247, 254).*

In addition, can the authors determine which layers of cortex were specifically affected by the insomnia (if such is possible).

*Although it is a fascinating question, unfortunately, the Cortical Thickness analyses cannot determine which cortical layers are affected.*

6. It would be helpful if the authors could explain how the numbers were utilized in measurement of the structures described in the MRI's.

*To perform the statistical analyses in MRI studies, there is a pre-processing step of the MRI-scans, which is mandatory, including the normalization of the images. This step allows the comparability of Cortical Thickness measures between different MRI-scans, limiting inter-individual variability because of other factors such as total brain volume. Afterward, with a neuroimage-statistical software (Matlab, toolbox Cat12) we build a statistical model (correlation or t-test in this case) including all the normalized MRI-scans and the row data of the variables of interest. These include the primary variable, ISI score (0-28) for the correlation analyses, or presence of insomnia (0-1) for the t-test. We then include the covariates, which include the basic ones for MRI-analyses [gender (0-1), age (60-95), handedness (0-1), scan type (0-3)] and, other factors more hypothesis-oriented to make sure there is no extra bias, such as the GDS (0-20) or BA (0-2) in our study. The software runs the algorithm (correlation or t-test adjusting by the indicated factors) based on the statistical model indicated. It provides the output by highlighting on a surface brain map the areas with statistical significance. The correlation coefficient or the t-value are expressed as a color scale to visualize the strength of the correlation/t-test comparison in the highlighted areas. Since these analyses are not volumetry analyses but cortical thickness analyses, the figures are displayed as surface maps, not volumetry comparisons. We hope the present explanation is clarifying.*
