# Supplementary material for: Specific cortical and subcortical grey matter regions are associated with insomnia severity
Source: PLoS One. 2021 May 26;16(5):e0252076. doi: 10.1371/journal.pone.0252076 (PMC8153469; doi:10.1371/journal.pone.0252076)
Supplement: Attachment — Submitted filename: Response to reviewers .docx [file pone.0252076.s004.docx]

We thank the reviewers for their time and insightful comments for our manuscript: “Specific cortical and subcortical grey matter regions are associated with insomnia severity” (PONE-D-20-36484). We appreciate both the editor’s approval and Reviewer ‘s effort to address some important, clarifying elements about the manuscript.

**Response to Reviewer 1:**

My initial comments have been addressed by the authors, and I appreciate their revisions and the contribution of their work to the literature. With the revised manuscript, I have several additional suggestions.

1. First, I appreciate the t-tests between those with insomnia symptoms and those without. However, it appears that the cut-off values supplied in the text have incorrect direction of > and <. It should read that the insomnia group has an ISI >7 and that the non-insomnia group has an ISI < 8.

*Thank you for catching this typo, which is key for understanding the classification criteria used for insomnia and non-insomnia groups. We have now addressed it throughout the manuscript text, describing insomnia group as* *ISI≥8 and non-insomnia groups as* *ISI ≤7.*

2. Second, in regards to the groupings above, I prefer the terms insomnia and non-insomnia group versus insomniacs and non-insomniacs as the later could refer to more of those meeting or not meeting criteria for an insomnia disorder. Specific diagnostic information was not collected in this sample, so careful use of terms best reflects the nature of the sample to the reader.

*We agree with the reviewer that additional clinical data should be assessed to properly categorize the subjects as insomniac vs non-insomniac. According to the reviewer’s proposal, we have changed the terminology to ‘insomnia/non-insomnia groups’.*

3. Along theses lines, I appreciate the idea of this study relating cortical thickness to insomnia symptoms; however, the use of the term "sleep disorder" is misleading, again due to lack of diagnostic information within this sample.

*Thank you for raising this point. We have changed the sleep disorder term to ‘insomnia symptoms’ (line 299).*

4. Finally, a very small edit is suggesting in referring to the DSM-5 and not the DSM-V.

*Thank you for this suggestion, we have now corrected it.*
