# Supplementary material for: Specific cortical and subcortical grey matter regions are associated with insomnia severity
Source: PLoS One. 2021 May 26;16(5):e0252076. doi: 10.1371/journal.pone.0252076 (PMC8153469; doi:10.1371/journal.pone.0252076)
Supplement: Peer review history

# Peer review: Decision Letter 0

**DOI:** 10.1371/journal.pone.0252076.r001

**Authors:** Allan Siegel

22 Jan 2021

PONE-D-20-36484

Specific cortical and subcortical grey matter regions are associated with insomnia severity

PLOS ONE

Dear Dr. Walsh,

Thank you for submitting your manuscript to PLOS ONE. After careful consideration, we feel that it has merit but does not fully meet PLOS ONE’s publication criteria as it currently stands. Therefore, we invite you to submit a revised version of the manuscript that addresses the points raised during the review process.

In addition to the comments raised by Reviewer #1, I have a number of issues that I believe should be addressed to enhance the quality of the manuscript. These are indicated below:

1. The authors make an important observation relating cortical thickness to a sleep disorder and this point should be further underscored in the presentation of the revised manuscript.

2. The possible significance of brain cell alterations in the putamen are not sufficiently (if at all) described and explained. For example, what cortical inputs to the putamen could account for such changes? Moreover, in view of its potential importance, it would be helpful to show an MRI indicating how the putamen in question compares to a normal (control) individual. In addition, the authors should make an effort to further explain why or how sleep loss would affect specific regions of cortex and not others.

3. It would be helpful if the authors included a control group of subjects with their MRI's (i.e., subjects without sleep disorders but of the same age, medical treatment history, etc.). Otherwise, one may have no idea whether variations in the appearance of the MRI's might not represent normal variations among individuals, especially of middle age and beyond. Such comparisons would strengthen the reliability of the observations.

4. In addition to the correlational analyses, I am curious why the study was not designed to utilize t-tests or ANOVA's to compare the groups in question with appropriate control groups. I believe that such analyses would strengthen the manuscript.

5. It is very difficult to discern from the MRI's shown in the manuscript how one group differs from another. That is why specific comparisons between groups would be helpful. In addition, can the authors determine which layers of cortex were specifically affected by the insomnia (if such is possible).

6. It would be helpful if the authors could explain how the numbers were utilized in measurement of the structures described in the MRI's.

Please submit your revised manuscript by Mar 05 2021 11:59PM. If you will need more time than this to complete your revisions, please reply to this message or contact the journal office at plosone@plos.org. When you're ready to submit your revision, log on to https://www.editorialmanager.com/pone/ and select the 'Submissions Needing Revision' folder to locate your manuscript file.

Please include the following items when submitting your revised manuscript:

- A rebuttal letter that responds to each point raised by the academic editor and reviewer(s). You should upload this letter as a separate file labeled 'Response to Reviewers'.
- A marked-up copy of your manuscript that highlights changes made to the original version. You should upload this as a separate file labeled 'Revised Manuscript with Track Changes'.
- An unmarked version of your revised paper without tracked changes. You should upload this as a separate file labeled 'Manuscript'.

If you would like to make changes to your financial disclosure, please include your updated statement in your cover letter. Guidelines for resubmitting your figure files are available below the reviewer comments at the end of this letter.

If applicable, we recommend that you deposit your laboratory protocols in protocols.io to enhance the reproducibility of your results. Protocols.io assigns your protocol its own identifier (DOI) so that it can be cited independently in the future. For instructions see: http://journals.plos.org/plosone/s/submission-guidelines#loc-laboratory-protocols

We look forward to receiving your revised manuscript.

Kind regards,

Allan Siegel

Academic Editor

PLOS ONE

Journal Requirements:

When submitting your revision, we need you to address these additional requirements.

1. Please ensure that your manuscript meets PLOS ONE's style requirements, including those for file naming. The PLOS ONE style templates can be found at

https://journals.plos.org/plosone/s/file?id=wjVg/PLOSOne_formatting_sample_main_body.pdf and

https://journals.plos.org/plosone/s/file?id=ba62/PLOSOne_formatting_sample_title_authors_affiliations.pdf

2. We note that you have indicated that data from this study are available upon request. PLOS only allows data to be available upon request if there are legal or ethical restrictions on sharing data publicly. For information on unacceptable data access restrictions, please see http://journals.plos.org/plosone/s/data-availability#loc-unacceptable-data-access-restrictions.

In your revised cover letter, please address the following prompts:

a) If there are ethical or legal restrictions on sharing a de-identified data set, please explain them in detail (e.g., data contain potentially identifying or sensitive patient information) and who has imposed them (e.g., an ethics committee). Please also provide contact information for a data access committee, ethics committee, or other institutional body to which data requests may be sent.

b) If there are no restrictions, please upload the minimal anonymized data set necessary to replicate your study findings as either Supporting Information files or to a stable, public repository and provide us with the relevant URLs, DOIs, or accession numbers. Please see http://www.bmj.com/content/340/bmj.c181.long for guidelines on how to de-identify and prepare clinical data for publication. For a list of acceptable repositories, please see http://journals.plos.org/plosone/s/data-availability#loc-recommended-repositories.

We will update your Data Availability statement on your behalf to reflect the information you provide.

3. Please remove your figures from within your manuscript file, leaving only the individual TIFF/EPS image files, uploaded separately.  These will be automatically included in the reviewers’ PDF.

[Note: HTML markup is below. Please do not edit.]

Reviewers' comments:

Reviewer's Responses to Questions

**Comments to the Author**

1. Is the manuscript technically sound, and do the data support the conclusions?

The manuscript must describe a technically sound piece of scientific research with data that supports the conclusions. Experiments must have been conducted rigorously, with appropriate controls, replication, and sample sizes. The conclusions must be drawn appropriately based on the data presented. 

Reviewer #1: Partly

**********

2. Has the statistical analysis been performed appropriately and rigorously? 

Reviewer #1: Yes

**********

3. Have the authors made all data underlying the findings in their manuscript fully available?

The PLOS Data policy requires authors to make all data underlying the findings described in their manuscript fully available without restriction, with rare exception (please refer to the Data Availability Statement in the manuscript PDF file). The data should be provided as part of the manuscript or its supporting information, or deposited to a public repository. For example, in addition to summary statistics, the data points behind means, medians and variance measures should be available. If there are restrictions on publicly sharing data—e.g. participant privacy or use of data from a third party—those must be specified.

Reviewer #1: Yes

**********

4. Is the manuscript presented in an intelligible fashion and written in standard English?

PLOS ONE does not copyedit accepted manuscripts, so the language in submitted articles must be clear, correct, and unambiguous. Any typographical or grammatical errors should be corrected at revision, so please note any specific errors here.

Reviewer #1: Yes

**********

5. Review Comments to the Author

Please use the space provided to explain your answers to the questions above. You may also include additional comments for the author, including concerns about dual publication, research ethics, or publication ethics. (Please upload your review as an attachment if it exceeds 20,000 characters)

Reviewer #1: The current study examines the relationship between insomnia severity and grey matter regions in a sample of 120 healthy older adults while adjusting for depressive symptoms and likelihood of sleep apnea. Grey matter regions were assessed by 3T brain MRI. Insomnia severity, depressive symptoms, and likelihood of sleep apnea were assessed via validated, self-report questionnaire. After adjusting for depressive symptoms, results revealed that greater insomnia severity was significantly related to decreased cortical thickness in the right ventral orbitofrontal area, right temporo-parietal junction, and left insula. Adjustment for the likelihood of sleep apnea did not impact any of these relationships. None of these relationships survived FWE multiple comparison correction. Additionally, greater insomnia severity was related to higher left putamen volume, surviving FDR multiple comparisons. Neither the adjustment for the likelihood of sleep apnea, nor for depressive symptoms impacted this result. The authors discuss the implications of these findings in healthy older adults and call for longitudinal investigation to determine how these results align with dementia risk.

This area of study is important. Strengths of the study include a well-defined healthy group of older adults who underwent standardized procedures, consideration of depressive symptoms and likelihood of apnea as co-variates, and relation of the current findings to the broader literature in the discussion section. The study could also be strengthened further, including the following:

(1) Of all self-report measures, the likelihood of sleep apnea presents the greatest concern as both insomnia severity and depressive symptoms are diagnosed and treated based on self-report. It could be helpful to better articulate this limitation in the discussion section, and the need for future studies to assess actual presence of apnea via in-lab or at-home overnight testing.

(2) The percentage of individuals with likelihood of apnea seems to be fairly similar to other published healthy older adult samples. However, the insomnia severity and potentially the depressive symptom averages appear lower than usual. It could be helpful to not only provide the average for these measures, but percentage of individuals within each cut-off range. For example, it could be helpful to provide the number of individuals in the “non-insomnia,” “sub-threshold insomnia,” and “moderate insomnia” categories as well as more fully acknowledge the potential differences in lower rates of symptoms in the current sample in the discussion section more fully.

(3) Many of the results do not survive corrections for multiple comparisons. It could be helpful to discuss this more fully in the discussion section and insure it is represented in the study abstract.

(4) There is possibly a typo in the results sections under the title, “Associations between ISI scores and GM regions”. There is mention of right medial orbitofrontal regions of significance, whereas, otherwise in the paper there is mention of right ventral orbitofrontal regions of significance.

(5) Was this a planned analysis prior to data collection or an exploratory analysis with existing data? It could be helpful to the reader to understand the degree of exploratory approach for the current paper.

**********

6. PLOS authors have the option to publish the peer review history of their article (what does this mean?). If published, this will include your full peer review and any attached files.

If you choose “no”, your identity will remain anonymous but your review may still be made public.

**Do you want your identity to be public for this peer review?** For information about this choice, including consent withdrawal, please see our Privacy Policy.

Reviewer #1: No

[NOTE: If reviewer comments were submitted as an attachment file, they will be attached to this email and accessible via the submission site. Please log into your account, locate the manuscript record, and check for the action link "View Attachments". If this link does not appear, there are no attachment files.]

While revising your submission, please upload your figure files to the Preflight Analysis and Conversion Engine (PACE) digital diagnostic tool, https://pacev2.apexcovantage.com/. PACE helps ensure that figures meet PLOS requirements. To use PACE, you must first register as a user. Registration is free. Then, login and navigate to the UPLOAD tab, where you will find detailed instructions on how to use the tool. If you encounter any issues or have any questions when using PACE, please email PLOS at figures@plos.org. Please note that Supporting Information files do not need this step.

# Author comment: Author response to Decision Letter 0

**DOI:** 10.1371/journal.pone.0252076.r002

12 Apr 2021

We thank the reviewers for their time and insightful comments for our manuscript: “Specific cortical and subcortical grey matter regions are associated with insomnia severity” (PONE-D-20-36484). We appreciate Reviewer 1 highlighting the importance of the study and the resulting manuscript. We have addressed both the Reviewer’s and Editor’s comments in Response to reviewers document and in the manuscript (see the attached document for the response to reviewer text and images). We hope they alleviate any of your prior concerns. Your comments have improved the clarity of our manuscript.

Response to reviewers:

Reviwer #1:

1. Of all self-report measures, the likelihood of sleep apnea presents the greatest concern as both insomnia severity and depressive symptoms are diagnosed and treated based on self-report. It could be helpful to better articulate this limitation in the discussion section, and the need for future studies to assess actual presence of apnea via in-lab or at-home overnight testing.

Thank you for pointing this out. We agree with the reviewer that determining the presence of sleep apnea with objective measures would increase the accuracy of this measurement. We have stressed this limitation in the discussion section and highlighted the need for further studies using objective tools for apnea testing. 

Line 463-464: Therefore, further studies assessing the presence of sleep apnea with objective (at-home or in-lab) diagnostic methods are needed.

2. The percentage of individuals with likelihood of apnea seems to be fairly similar to other published healthy older adult samples. However, the insomnia severity and potentially the depressive symptom averages appear lower than usual. It could be helpful to not only provide the average for these measures, but percentage of individuals within each cut-off range. For example, it could be helpful to provide the number of individuals in the “non-insomnia,” “sub-threshold insomnia,” and “moderate insomnia” categories as well as more fully acknowledge the potential differences in lower rates of symptoms in the current sample in the discussion section more fully. 

We agree that providing the categorical distribution of the ISI could be helpful for the reader to better understand the sample characteristics. We have now included the percentage of subjects in each of the categories in Table 1. Furthermore, we have stressed the fact that the subjects included scored mostly in the “no clinically significant insomnia” or “subthreshold insomnia” categories in the discussion section. Moreover, following the reviewer suggestion, we have included the discussion of potential differences in insomnia prevalence between other studies and ours (lines 308-319). 

3. Many of the results do not survive corrections for multiple comparisons. It could be helpful to discuss this more fully in the discussion section and insure it is represented in the study abstract. 

Although it is common in studies of healthy aging utilizing MRI-imaging to observe findings not surviving multiple comparisons due to the small effect size, we fully agree with the reviewer that it is important to highlight this because of its potential implications. We have now addressed this point by extending the statement in the discussion (lines 468-471). Regarding the abstract, we apologize if that was not clear enough by reporting the results as p<0.001, uncorrected FWE. We have now included the statement ‘The results were no longer significant following FWE multiple comparisons’ (Lines 51-52).

4. There is possibly a typo in the results sections under the title, “Associations between ISI scores and GM regions”. There is mention of right medial orbitofrontal regions of significance, whereas, otherwise in the paper there is mention of right ventral orbitofrontal regions of significance.

Thank you for catching this. It was a typo which we have now corrected. 

5. Was this a planned analysis prior to data collection or an exploratory analysis with existing data? It could be helpful to the reader to understand the degree of exploratory approach for the current paper.

This was a hypothesis driven analysis of existing data. Dr. Walsh was interested in better understanding the association between disrupted sleep and brain health in older adults. Dr. Falgàs with her interests in brain imaging and sleep disruption raised came to Dr. Walsh with her hypotheses in this area and assessed the existing dataset. 

Line 100-101: Therefore, in the current study we utilize an existing dataset to assess the association of both cortical and subcortical GM regions to perceived insomnia severity in a cohort of healthy older adults, taking into account the potential effect of depression and sleep apnea.

Editor comments:

1. The authors make an important observation relating cortical thickness to a sleep disorder and this point should be further underscored in the presentation of the revised manuscript. 

Thank you for raising this point; we have now addressed the manuscript to highlight this relationship in the manuscript further (305-306, 441-446).

2. The possible significance of brain cell alterations in the putamen are not sufficiently (if at all) described and explained. For example, what cortical inputs to the putamen could account for such changes? Moreover, in view of its potential importance, it would be helpful to show an MRI indicating how the putamen in question compares to a normal (control) individual. In addition, the authors should make an effort to further explain why or how sleep loss would affect specific regions of cortex and not others.

We have now extended the explanation on the putamen’s relevance and cited the corresponding literature. 

Lines 420-427: The putamen, as a main component of the striatum, is highly interconnected with frontal cortical areas such as the orbitofrontal and prefrontal regions. The connectivity between these cortico-subcortical regions shapes the frontostriatal circuit, which has been related to sleep-wake regulation. Although the mechanism by which subcortical areas, such as the putamen, regulate arousal is still poorly understood, the dysfunction of the frontostriatal circuit seems to have a role in insomnia. In this line, prior functional MRI studies have reported altered patterns of connectivity between subcortical (putamen) and cortical (frontal) regions in insomnia patients [52-54].

Lu FM et al., Disrupted Topology of Frontostriatal Circuits Is Linked to the Severity of Insomnia. Front Neurosci. 2017 Apr 19;11:214. doi: 10.3389/fnins.2017.00214. 

Zhou F et al. Altered long- and short-range functional connectivity density associated with poor sleep quality in patients with chronic insomnia disorder: A resting-state fMRI study. Brain Behav. 2020 Nov;10(11):e01844. doi: 10.1002/brb3.1844. 

Zou G, et al. Altered thalamic connectivity in insomnia disorder during wakefulness and sleep. Hum Brain Mapp. 2021 Jan;42(1):259-270. doi: 10.1002/hbm.25221. 

When performing MRI-analyses, different factors can contribute to the final measure of the putamen volumes. Because of that, when evaluating the differences in a correlation model or t-test, we control for factors such as Total Intracranial Volume, gender, age, handedness, etc. Although we could provide the image of an MRI-scan of a random patient and control (see below), we consider this comparison not accurate enough to be included in the manuscript. We believe the visual inspection of the putamen on an MRI could be misleading since the direct comparison of the MRI-scan would not be reflecting the complexity of the whole analyses, and it represents a non-adjusted output. However, to provide further information regarding the putamen volumes in insomnia at the group level, we have included Supporting Information Figure 2 (S2 Fig), the boxplots comparing the normalized putamen volumes in insomnia and non-insomnia subjects. 

S2 Fig. Putamen volumes between insomniacs and non-insomniacs. Box plot showing the mean of the normalized volumes of right and left putamen in insomnia (ISI<7) and non-insomnia (ISI≤8) subjects (n=120). *Significance p<0.05

3. It would be helpful if the authors included a control group of subjects with their MRI's (i.e., subjects without sleep disorders but of the same age, medical treatment history, etc.). Otherwise, one may have no idea whether variations in the appearance of the MRI's might not represent normal variations among individuals, especially of middle age and beyond. Such comparisons would strengthen the reliability of the observations.

Thank you for bringing this up. The present study aims to evaluate the correlation of the spectrum of insomnia severity (using ISI, which scores from 0 to 28) and Cortical Thickness in a cohort of healthy older adults. The reason for this approach is that prior MRI studies have already used a case-control study design (insomniacs vs. controls) (Grau-Rivera et al., 2020; Altena et al., 2010; Sexton et al., 2014). Although these studies have varying results, overall, they highlight a detrimental effect of insomnia or low sleep quality on the orbitofrontal and prefrontal areas. However, insomnia symptoms occur on a spectrum, and focusing on cut-offs or thresholds of insomnia being present vs not present miss out on the importance of addressing even low-level insomnia, which appear to relate to brain changes as well. Therefore, we believe there is an unmet gap in evaluating how the spectrum of insomnia (including symptoms below the insomnia category or mild insomnia symptoms) affects brain changes in healthy aging which we are addressing in this manuscript. 

The CTh-neuroimaging analyses performed in the present study include the standard normalization step of the MRI-scans to a template during the pre-processing of the images that adjust and, therefore, limit the potential contribution of interindividual variations. Additionally, the regression model adjusted the data by other factors that could potentially influence the results: age, gender, scan type, and handedness. Furthermore, we included GDS (depression scale) and BA (apnea likelihood) because they are frequently associated with insomnia. 

In order to address your concerns, we have included a comparison of insomniacs (ISI<7) vs non-insomniacs (ISI≤8) in the Supporting Information (S1 Fig).

S2 Fig. Cortical Thickness differences between insomniacs and non-insomniacs, adjusted by Geriatric Depression Scale. Cortical thickness t-test comparison between insomnia (ISI<7) and non-insomnia (ISI≤8) groups (n=119) adjusting by age, sex, handedness, scan type and Geriatric Depression Scale. Only regions with P-value <.001 (uncorrected FWE) are shown. T-values are expressed as a color scale.

Grau-Rivera O, et al. Association between insomnia and cognitive performance, gray matter volume, and white matter microstructure in cognitively unimpaired adults. Alzheimers Res Ther. 2020;12(1):4. Published 2020 Jan 7. doi:10.1186/s13195-019-0547-3. 

Altena E, et al. Reduced orbitofrontal and parietal gray matter in chronic insomnia: a voxel-based morphometric study. Biol Psychiatry. 2010 Jan 15;67(2):182-5. doi: 10.1016/j.biopsych.2009.08.003.

Sexton CE, et al. Poor sleep quality is associated with increased cortical atrophy in community-dwelling adults. Neurology. 2014;83(11):967–973. doi:10.1212/WNL.0000000000000774. 

4. In addition to the correlational analyses, I am curious why the study was not designed to utilize t-tests or ANOVA's to compare the groups in question with appropriate control groups. I believe that such analyses would strengthen the manuscript.

The study's design was based on the objective of evaluating the correlation of the spectrum of insomnia severity and Cortical Thickness in a cohort of healthy older adults. We used a cohort of older adults enriched by individuals not reaching the insomnia diagnosis level or having mild insomnia to test its correlation with brain Cortical Thickness. We apologize if the correlation results are not clear enough. In order to provide more clarity and to address your concerns, we have performed additional analyses dividing the cohort into two groups: insomnia and controls (non-insomnia). The results of the additional analyses, shown in the S1 Fig, indicate the t-value on the regions showing a statistical difference in Cortical Thickness between insomnia and non-insomnia groups. Similar to the correlation analyses, the CTh t-test comparisons between insomnia and non-insomnia groups showed lower CTh in certain orbitofrontal, prefrontal and temporo-parietal areas (p<0.001, uncorrected). However, differences in the insula were not replicated (lines 239-242, 401-403).

5. It is very difficult to discern from the MRI's shown in the manuscript how one group differs from another. That is why specific comparisons between groups would be helpful

The brain surface maps shown in figures 2, 3 and Supporting Information Fig 1, are the standard output obtained by the MRI-Cortical Thickness analysis. In Figure 2 and 3, brain maps show the areas on which the correlation between ISI and CTh is statistically significant. In Supplemental Fig 1, brain maps show the areas on which the t-test analyses comparing CTh measures between case (insomnia) and controls (non-insomniacs) are statistically significant. The color scale indicates the strength of the effect, indicating the correlation coefficient for the correlation analyses and the t-value for the t-test. Thank you for bringing this up so we had the opportunity to more fully explain how to interpret the figures in the figure legends; we hope the analyses output are now clearer for the reader (lines 247, 254). 

 In addition, can the authors determine which layers of cortex were specifically affected by the insomnia (if such is possible).

Although it is a fascinating question, unfortunately, the Cortical Thickness analyses cannot determine which cortical layers are affected. 

6. It would be helpful if the authors could explain how the numbers were utilized in measurement of the structures described in the MRI's.

To perform the statistical analyses in MRI studies, there is a pre-processing step of the MRI-scans, which is mandatory, including the normalization of the images. This step allows the comparability of Cortical Thickness measures between different MRI-scans, limiting inter-individual variability because of other factors such as total brain volume. Afterward, with a neuroimage-statistical software (Matlab, toolbox Cat12) we build a statistical model (correlation or t-test in this case) including all the normalized MRI-scans and the row data of the variables of interest. These include the primary variable, ISI score (0-28) for the correlation analyses, or presence of insomnia (0-1) for the t-test. We then include the covariates, which include the basic ones for MRI-analyses [gender (0-1), age (60-95), handedness (0-1), scan type (0-3)] and, other factors more hypothesis-oriented to make sure there is no extra bias, such as the GDS (0-20) or BA (0-2) in our study. The software runs the algorithm (correlation or t-test adjusting by the indicated factors) based on the statistical model indicated. It provides the output by highlighting on a surface brain map the areas with statistical significance. The correlation coefficient or the t-value are expressed as a color scale to visualize the strength of the correlation/t-test comparison in the highlighted areas. Since these analyses are not volumetry analyses but cortical thickness analyses, the figures are displayed as surface maps, not volumetry comparisons. We hope the present explanation is clarifying.

Attachment Submitted filename: Response to reviewers .docx Click here for additional data file.

# Peer review: Decision Letter 1

**DOI:** 10.1371/journal.pone.0252076.r003

**Authors:** Allan Siegel

4 May 2021

PONE-D-20-36484R1

Specific cortical and subcortical grey matter regions are associated with insomnia severity

PLOS ONE

Dear Dr. Walsh,

Thank you for submitting your manuscript to PLOS ONE. Overall, you have responded effectively to the comments of the reviewers including myself as editor and these major concerns expressed in our original response are no longer an issue. However, reviewer #1 has a few additional issues  that need to be resolved. (See below for the specific comments raised by Reviewer #1). Therefore, we invite you to submit a revised version of the manuscript at your earliest convenience that addresses these points raised by this reviewer. 

Please submit your revised manuscript by Jun 18 2021 11:59PM. If you will need more time than this to complete your revisions, please reply to this message or contact the journal office at plosone@plos.org. When you're ready to submit your revision, log on to https://www.editorialmanager.com/pone/ and select the 'Submissions Needing Revision' folder to locate your manuscript file.

Please include the following items when submitting your revised manuscript:

- A rebuttal letter that responds to each point raised by the academic editor and reviewer(s). You should upload this letter as a separate file labeled 'Response to Reviewers'.
- A marked-up copy of your manuscript that highlights changes made to the original version. You should upload this as a separate file labeled 'Revised Manuscript with Track Changes'.
- An unmarked version of your revised paper without tracked changes. You should upload this as a separate file labeled 'Manuscript'.

If you would like to make changes to your financial disclosure, please include your updated statement in your cover letter. Guidelines for resubmitting your figure files are available below the reviewer comments at the end of this letter.

If applicable, we recommend that you deposit your laboratory protocols in protocols.io to enhance the reproducibility of your results. Protocols.io assigns your protocol its own identifier (DOI) so that it can be cited independently in the future. For instructions see: http://journals.plos.org/plosone/s/submission-guidelines#loc-laboratory-protocols. Additionally, PLOS ONE offers an option for publishing peer-reviewed Lab Protocol articles, which describe protocols hosted on protocols.io. Read more information on sharing protocols at https://plos.org/protocols?utm_medium=editorial-email&utm_source=authorletters&utm_campaign=protocols.

We look forward to receiving your revised manuscript.

Kind regards,

Allan Siegel

Academic Editor

PLOS ONE

Journal Requirements:

Please review your reference list to ensure that it is complete and correct. If you have cited papers that have been retracted, please include the rationale for doing so in the manuscript text, or remove these references and replace them with relevant current references. Any changes to the reference list should be mentioned in the rebuttal letter that accompanies your revised manuscript. If you need to cite a retracted article, indicate the article’s retracted status in the References list and also include a citation and full reference for the retraction notice.

[Note: HTML markup is below. Please do not edit.]

Reviewers' comments:

Reviewer's Responses to Questions

**Comments to the Author**

1. If the authors have adequately addressed your comments raised in a previous round of review and you feel that this manuscript is now acceptable for publication, you may indicate that here to bypass the “Comments to the Author” section, enter your conflict of interest statement in the “Confidential to Editor” section, and submit your "Accept" recommendation.

Reviewer #1: (No Response)

**********

2. Is the manuscript technically sound, and do the data support the conclusions?

The manuscript must describe a technically sound piece of scientific research with data that supports the conclusions. Experiments must have been conducted rigorously, with appropriate controls, replication, and sample sizes. The conclusions must be drawn appropriately based on the data presented. 

Reviewer #1: Yes

**********

3. Has the statistical analysis been performed appropriately and rigorously? 

Reviewer #1: Yes

**********

4. Have the authors made all data underlying the findings in their manuscript fully available?

The PLOS Data policy requires authors to make all data underlying the findings described in their manuscript fully available without restriction, with rare exception (please refer to the Data Availability Statement in the manuscript PDF file). The data should be provided as part of the manuscript or its supporting information, or deposited to a public repository. For example, in addition to summary statistics, the data points behind means, medians and variance measures should be available. If there are restrictions on publicly sharing data—e.g. participant privacy or use of data from a third party—those must be specified.

Reviewer #1: Yes

**********

5. Is the manuscript presented in an intelligible fashion and written in standard English?

PLOS ONE does not copyedit accepted manuscripts, so the language in submitted articles must be clear, correct, and unambiguous. Any typographical or grammatical errors should be corrected at revision, so please note any specific errors here.

Reviewer #1: Yes

**********

6. Review Comments to the Author

Please use the space provided to explain your answers to the questions above. You may also include additional comments for the author, including concerns about dual publication, research ethics, or publication ethics. (Please upload your review as an attachment if it exceeds 20,000 characters)

Reviewer #1: My initial comments have been addressed by the authors, and I appreciate their revisions and the contribution of their work to the literature. With the revised manuscript, I have several additional suggestions.

First, I appreciate the t-tests between those with insomnia symptoms and those without. However, it appears that the cut-off values supplied in the text have incorrect direction of > and <. It should read that the insomnia group has an ISI >7 and that the non-insomnia group has an ISI < 8.

Second, in regards to the groupings above, I prefer the terms insomnia and non-insomnia group versus insomniacs and non-insomniacs as the later could refer to more of those meeting or not meeting criteria for an insomnia disorder. Specific diagnostic information was not collected in this sample, so careful use of terms best reflects the nature of the sample to the reader. Along theses lines, I appreciate the idea of this study relating cortical thickness to insomnia symptoms; however, the use of the term "sleep disorder" is misleading, again due to lack of diagnostic information within this sample.

Finally, a very small edit is suggesting in referring to the DSM-5 and not the DSM-V.

**********

7. PLOS authors have the option to publish the peer review history of their article (what does this mean?). If published, this will include your full peer review and any attached files.

If you choose “no”, your identity will remain anonymous but your review may still be made public.

**Do you want your identity to be public for this peer review?** For information about this choice, including consent withdrawal, please see our Privacy Policy.

Reviewer #1: No

[NOTE: If reviewer comments were submitted as an attachment file, they will be attached to this email and accessible via the submission site. Please log into your account, locate the manuscript record, and check for the action link "View Attachments". If this link does not appear, there are no attachment files.]

While revising your submission, please upload your figure files to the Preflight Analysis and Conversion Engine (PACE) digital diagnostic tool, https://pacev2.apexcovantage.com/. PACE helps ensure that figures meet PLOS requirements. To use PACE, you must first register as a user. Registration is free. Then, login and navigate to the UPLOAD tab, where you will find detailed instructions on how to use the tool. If you encounter any issues or have any questions when using PACE, please email PLOS at figures@plos.org. Please note that Supporting Information files do not need this step.

# Author comment: Author response to Decision Letter 1

**DOI:** 10.1371/journal.pone.0252076.r004

6 May 2021

We thank the reviewers for their time and insightful comments for our manuscript: “Specific cortical and subcortical grey matter regions are associated with insomnia severity” (PONE-D-20-36484). We appreciate both the editor’s approval and Reviewer ‘s effort to address some important, clarifying elements about the manuscript. 

Response to Reviewer 1:

My initial comments have been addressed by the authors, and I appreciate their revisions and the contribution of their work to the literature. With the revised manuscript, I have several additional suggestions.

1. First, I appreciate the t-tests between those with insomnia symptoms and those without. However, it appears that the cut-off values supplied in the text have incorrect direction of > and <. It should read that the insomnia group has an ISI >7 and that the non-insomnia group has an ISI < 8.

Thank you for catching this typo, which is key for understanding the classification criteria used for insomnia and non-insomnia groups. We have now addressed it throughout the manuscript text, describing insomnia group as ISI≥8 and non-insomnia groups as ISI ≤7.

2. Second, in regards to the groupings above, I prefer the terms insomnia and non-insomnia group versus insomniacs and non-insomniacs as the later could refer to more of those meeting or not meeting criteria for an insomnia disorder. Specific diagnostic information was not collected in this sample, so careful use of terms best reflects the nature of the sample to the reader. 

We agree with the reviewer that additional clinical data should be assessed to properly categorize the subjects as insomniac vs non-insomniac. According to the reviewer’s proposal, we have changed the terminology to ‘insomnia/non-insomnia groups’.

3. Along theses lines, I appreciate the idea of this study relating cortical thickness to insomnia symptoms; however, the use of the term "sleep disorder" is misleading, again due to lack of diagnostic information within this sample.

Thank you for raising this point. We have changed the sleep disorder term to ‘insomnia symptoms’ (line 299).

4. Finally, a very small edit is suggesting in referring to the DSM-5 and not the DSM-V.

Thank you for this suggestion, we have now corrected it.

Attachment Submitted filename: Response to reviewers .docx Click here for additional data file.

# Editor report: Decision Letter 2

**DOI:** 10.1371/journal.pone.0252076.r005

**Authors:** Allan Siegel

10 May 2021

Specific cortical and subcortical grey matter regions are associated with insomnia severity

PONE-D-20-36484R2

Dear Dr. Walsh,

We’re pleased to inform you that your revised manuscript has been judged scientifically suitable for publication and will be formally accepted for publication once it meets all outstanding technical requirements.

Within one week, you’ll receive an e-mail detailing the required amendments. When these have been addressed, you’ll receive a formal acceptance letter and your manuscript will be scheduled for publication.

An invoice for payment will follow shortly after the formal acceptance. To ensure an efficient process, please log into Editorial Manager at http://www.editorialmanager.com/pone/, click the 'Update My Information' link at the top of the page, and double check that your user information is up-to-date. If you have any billing related questions, please contact our Author Billing department directly at authorbilling@plos.org.

If your institution or institutions have a press office, please notify them about your upcoming paper to help maximize its impact. If they’ll be preparing press materials, please inform our press team as soon as possible -- no later than 48 hours after receiving the formal acceptance. Your manuscript will remain under strict press embargo until 2 pm Eastern Time on the date of publication. For more information, please contact onepress@plos.org.

Kind regards,

Allan Siegel

Academic Editor

PLOS ONE

Additional Editor Comments (optional):

Reviewers' comments:

# Editor report: Acceptance letter

**DOI:** 10.1371/journal.pone.0252076.r006

**Authors:** Allan Siegel

17 May 2021

PONE-D-20-36484R2 

Specific cortical and subcortical grey matter regions are associated with insomnia severity. 

Dear Dr. Walsh:

I'm pleased to inform you that your manuscript has been deemed suitable for publication in PLOS ONE. Congratulations! Your manuscript is now with our production department. 

If your institution or institutions have a press office, please let them know about your upcoming paper now to help maximize its impact. If they'll be preparing press materials, please inform our press team within the next 48 hours. Your manuscript will remain under strict press embargo until 2 pm Eastern Time on the date of publication. For more information please contact onepress@plos.org.

If we can help with anything else, please email us at plosone@plos.org. 

Thank you for submitting your work to PLOS ONE and supporting open access. 

Kind regards, 

PLOS ONE Editorial Office Staff

on behalf of

Dr Allan Siegel 

Academic Editor

PLOS ONE
